# Comprehensive Genetic Analysis of Early Host Body Reactions to the Bioactive and Bio-Inert Porous Scaffolds

**DOI:** 10.1371/journal.pone.0085132

**Published:** 2014-01-14

**Authors:** Tomo Ehashi, Taro Takemura, Nobutaka Hanagata, Takashi Minowa, Hisatoshi Kobayashi, Kazuhiko Ishihara, Tetsuji Yamaoka

**Affiliations:** 1 Department of Biomedical Engineering, National Cerebral and Cardiovascular Center Research Institute, Osaka, Japan; 2 Core Research Evolutional Science and Technology (CREST), Japan Science and Technology Agency, Tokyo, Japan; 3 Nanotechnology Innovation Station, National Institute for Materials Science, Ibaraki, Japan; 4 Biomaterials Center, National Institute for Materials Science, Ibaraki, Japan; 5 Department of Materials Engineering and Department of Bioengineering, School of Engineering, The University of Tokyo, Tokyo, Japan; Osaka University, Japan

## Abstract

To design scaffolds for tissue regeneration, details of the host body reaction to the scaffolds must be studied. Host body reactions have been investigated mainly by immunohistological observations for a long time. Despite of recent dramatic development in genetic analysis technologies, genetically comprehensive changes in host body reactions are hardly studied. There is no information about host body reactions that can predict successful tissue regeneration in the future. In the present study, porous polyethylene scaffolds were coated with bioactive collagen or bio-inert poly(2-methacryloyloxyethyl phosphorylcholine-co-*n*-butyl methacrylate) (PMB) and were implanted subcutaneously and compared the host body reaction to those substrates by normalizing the result using control non-coat polyethylene scaffold. The comprehensive analyses of early host body reactions to the scaffolds were carried out using a DNA microarray assay. Within numerous genes which were expressed differently among these scaffolds, particular genes related to inflammation, wound healing, and angiogenesis were focused upon. Interleukin (IL)-1β and IL-10 are important cytokines in tissue responses to biomaterials because IL-1β promotes both inflammation and wound healing and IL-10 suppresses both of them. IL-1β was up-regulated in the collagen-coated scaffold. Collagen-specifically up-regulated genes contained both M1- and M2-macrophage-related genes. Marked vessel formation in the collagen-coated scaffold was occurred in accordance with the up-regulation of many angiogenesis-inducible factors. The DNA microarray assay provided global information regarding the host body reaction. Interestingly, several up-regulated genes were detected even on the very bio-inert PMB-coated surfaces and those genes include inflammation-suppressive and wound healing-suppressive IL-10, suggesting that not only active tissue response but also the inert response may relates to these genetic regulations.

## Introduction

The key features of biomaterials scaffolds which are attractive for tissue regenerations are unclear although numerous researchers have been investigating the biological reactions to various materials *in vitro* and *in vivo*. Cell responses are affected by structure, physical properties, and the chemical properties of the scaffold [1], [2]. For example, the size and shape of nano and microparticles modulate phagocytosis of macrophages, and mechanical strain as a physical property affects macrophage hydrolytic activation [3], [4]. It is known that not all chemically biocompatible materials are adequate for tissue regeneration. Hyaluronan is sometimes clinically used as post-operative tissue adhesion prevention matrices due to noncytotoxic and biodegradable [5], [6]. However, its hydrophilic surface property may disturb the cell adhesion and tissue regeneration when used as a scaffold.

Generally, tissue responses to biomaterials have been assessed by histological observations such as material encapsulation, which suggests a failure of tissue regeneration [7], [8]. The number and distribution of immune cells are also used to judge the material inadequacy [9]–[11]. Recently, the expression and production of cytokines from material-attached cells on various surfaces properties have been investigated *in vivo* and *in vitro* [12]–[15]. Brodbeck *et al*. have proposed a classification of cytokines into four groups according to their roles, *i.e.*, promotion and suppression of inflammation or wound healing [12]. They implanted materials with various surface properties to compare the influence on leukocyte cytokines expression, but suitable materials for tissue regeneration were not observed in that study.

In biomaterials implantation, monocytes and macrophages mainly conduct foreign body reactions [16]. When the immune system is stimulated by foreign bodies, macrophages polarize into the M1- and M2-types, which correspond to classical and alternative activation, respectively [17]–[19]. It has been reported that M2 polarization is favorable to remodeling in biological scaffolds [20]. However, no synthetic material was used to consider macrophage profiling and tissue regeneration.

Many research groups have measured the expression and production levels of cytokines by RT-PCR and ELISA, respectively [13]–[15]. Although RT-PCR techniques have allowed for easier and more sensitive measurement than ELISA, the range of measurable cytokines is limited. After completion of the genome project, molecular biological analysis has dramatically improved by DNA microarray techniques. There have been some studies using DNA microarray related to *in vitro* cellular responses to several biomaterials [21]–[24]. In these studies, the influences of material characteristics on cell apoptosis, metabolism, and maturation have been analyzed by various analytical methods. The significantly up-regulated and down-regulated genes among cells on different materials were picked-up and roughly classified based on their functions. However, it has never been suggested which responses of immune cells and other cells to materials are indispensable for tissue regeneration. Moreover, there has been no report on the comprehensive analysis for *in vivo* tissue reactions to biomaterials.

Needless to say, these tissue responses are highly depends on various features of implanted materials. Collagen is distributed everywhere in body. It is highly biocompatible and is going to use in clinical treatment as scaffold for such as nerve or cartridge regeneration [25], [26]. Collagen positively attracts host cells to attach on the surface and is degraded by enzyme in host body, indicating that collagen is one of the most bioactive substrates. On the other hands, poly(2-methacryloyloxyethyl phosphorylcholine) (MPC) disturbs protein adsorption and cell attachment owing to its high hydrophilic feature. MPC polymer is well known to be an useful materials for coating surface of implants such as artificial hearts, stents, or hip joints because the host cells do not attach on the MPC surface due to its bio-inertness [27], [28]. Collagen and MPC are one of the most representative models of facilitating tissue regeneration and being ignored by host body, respectively. Once bio-incompatible materials are implanted, terrible inflammation and materials encapsulation will be occur and such host body reactions will lead failure of tissue regeneration. Therefore, bio-incompatible materials in addition to bioactive and bio-inert materials should be assessed and compared to investigate effective host body reaction for tissue regeneration.

In addition to the matrix nature, microstructure are also attracting great attention as the factors affecting the tissue responses. Porous structures including its pore size of the scaffold has been attracting great attention because they affect the angiogenesis, encapsulation, and cellular migration toward the scaffolds [29]–[32]. These factors would be strongly related to the fate of tissue regeneration. For example, porous polyvinyl alcohol scaffolds induce higher density of microvessels and their density was dramatically affected by pore size [32]. In that study, vascular density around non porous scaffold was much lower than that in the native tissue.

In order to understand initial host body reactions towards successful tissue regeneration, not only histological information but also genetic information of infiltrating tissue is needed. In this study, 3-dimensional PE porous scaffolds with collagen (bioactive) and poly(MPC-*co*-*n*-butyl methacrylate (BMA)) (PMB) (bio-inert) coating were used and genetic level of host body reactions after 7 days implantation were analyzed. Local RNAs in infiltrating cells into the porous scaffolds was extracted using laser microdissection technique. The relationships between the expression levels of important genes for tissue regeneration on the collagen and MPC surface scaffolds are discussed in combination with histological results.

## Materials and Methods

### Ethics Statement

All animal experiments were conducted in accordance with the Guidelines for Animal Experiments established by the Ministry of Health, Labour and Welfare of Japan, and by the National Cerebral and Cardiovascular Center Research Institute, Japan. The protocol was approved by the Committee on the Ethics of Animal Experiments of the National Cerebral and Cardiovascular Center Research Institute (Permit Number: 009017).

### Preparation of Scaffolds

Porous polyethylene (PE) substrates (Sunfine®AQ–200 and –900, mean pore size  = 157 µm and 32 µm, respectively) as a scaffold, were kindly donated by Asahi Chemicals (Tokyo, Japan). A 2-mm thickness of the PE substrate was cut out by a punch of 6 mm in diameter, and the disc-shaped substrates were washed in acetone with sonification 3 times and dried completely in vacuo. To prepare the bioactive scaffold, type I collagen (Cellmatrix; Nitta Gelatin, Osaka, Japan) was coated on the PE substrate as below. The PE substrates were oxidized in ozone atmosphere for 60 min to make the surface hydrophilic. Immediately after oxidation, they were immersed in 0.3 mg/mL collagen aqueous solution for 4 h and were washed 3 times with double distilled water. To prepare the bio-inert scaffolds, the PMB was synthesized by free radical polymerization of MPC and BMA [33]–[37]. The mole fraction of the MPC unit was 0.30 and weight averaged molecular weight was 6.0×10^6^. The ethanol solution of the PMB (1.0%) was dropped on the washed PE substrate and dried overnight to obtain PMB-coated scaffold. A non-coated PE substrate was used as the control scaffold since it is unable to regenerate tissues. All scaffolds were prepared under sterilized conditions.

### Surface analysis of modified PE substrate

Coatings of collagen and PMB on the PE substrate were confirmed by the static water contact angle measurement, ATR-FTIR spectroscopy, and energy dispersive X-ray spectrometry (EDS). For contact angle measurements and ATR-FTIR, collagen- and PMB-coated PE films instead of porous PE scaffold were prepared by the same method as used for coating on the PE substrate. All kinds of films were soaked in water 1 h before measurement of the contact angles. The air contact angles on non-, collagen-, and PMB-coated film surfaces were measured in water and calculated as the water contact angles (Kyowa Interface Science, Saitama, Japan). The ATR-FTIR spectra were obtained in 64 scans over a range of 750–4500 cm^−1^ by using an FTIR analyzer (Spectrum GX-Ramman, Perkin Elmer, MA, USA). The polymer coating at the middle of scaffold was confirmed with EDS (JEOL, Tokyo, Japan). In brief, porous PE substrates coated with collagen and PMB were cut at the middle, coated with platinum, and then obtained EDS spectrum to confirm coating polymer existence on the scaffold skeleton.

### Implantation of scaffolds in animals

Scaffolds were immersed in sterile saline overnight before implantation, and were implanted subcutaneously in rats or mice. Briefly, under anesthesia with isoflurane inhalation, an incision approximately 10 mm long was made in dorsal skin, and scaffolds were inserted into each subcutaneous pocket. Eight-weeks-old male C57BL/6 mice (SLC Japan, Shizuoka, Japan) and 8∼10-weeks-old male Wistar rats (SLC) were used for comprehensive genetic analysis (n = 1) and immunohistochemistry (n = 3), respectively. These scaffolds were resected with surrounding tissue at 7 days after operation, and the early foreign body reaction to these scaffolds was assessed. Implanted period (7 days) were decided by reason of that differences of reaction to several kinds of materials were the most drastic at that period in preliminary studies.

### Comprehensive gene expression analysis

Seven days after surgery, mice dorsal skins were cut around each scaffold, embedded in the OCT compound, and rapidly frozen in liquid nitrogen. The scaffolds with tissue were sectioned at 20 µm thick using a cryostat (CM1850, Leica, Germany) and captured on films specially made for laser capture microdissection. Sections on films were dried and fixed with 95% ethanol and acetone at –20°C, and were immediately air-dried. Tissues within 100 µm of the scaffold were selectively cut by laser (LMD6000, Leica), collected in Isogen (Nippon Gene, Osaka, Japan) RNA isolation solution, and stored in the freezer until RNA isolation.

Over 1 µg of total RNAs was isolated according to the manufacturer’s protocol from approximately 15 sections of each sample tissue. The RNAs were amplified and labeled using the Amino Allyl Message dAmp II aRNA kit (Ambion, TX, USA) according to the manufacturer’s protocol. Each of 825 ng Cy-3 and Cy-5 labeled aRNAs were mixed and fragmented, and the samples were loaded on the microarray (Whole Mouse Genome Oligo Microarray Kit 4×44K, Agilent Technologies, CA, USA). The microarray was set in a chamber and the hybridization reaction was performed under 65°C for 17 h.

The microarray image was obtained with GenePix4000B (Axon Instruments, CA, USA) and was transferred into GenePix Pro 4.1 software (Axon Instruments) to automatically quantify the fluorescence intensity of each spot. The signal intensities of Cy3 and Cy5 were normalized using the locally weighted scatter plot smoothing (LOWESS) normalization method. The DNA microarray was hybridized twice in different Cy3 and Cy5 combinations. Expression of each gene is expressed as log_2_(*I*_PMB coat_/*I*_non coat_) and log_2_(*I*_collagen coat_/*I*_non coat_), which represent expression levels based on the intensity of fluorescence of collagen- or PMB-coated scaffold samples in comparison with that of the non-coated scaffold sample. Microarray data were deposited in the Gene Expression Omnibus (GEO) database (#GSE52053).

### Histology

Scaffolds implanted in rat subcutaneous tissue were resected with surrounding tissue, embedded in OCT compound (Sakura Finetek, Tokyo, Japan), and sectioned by cryostat. Hematoxylin and eosin staining and immunostaining for CD68, which was present on the macrophage, were performed. For immunostaining, anti-rat CD68 antibody (AbD Serotec, Oxford, UK) as the first antibody and horse radish peroxidase-conjugated anti-mouse IgG as the secondary antibody (DAKO, Glostrup, Denmark) were used. Stained samples were observed and photographed by Coolscope (Nikon, Tokyo, Japan). Encapsulation thickness around implanted scaffold was measured on picture of HE staining. In brief, three points of fibrous connective tissue existing as thin membrane between scaffold and host body wall were measured on picture. Average encapsulation thickness was calculated and each value was statistically compared with the thickness of non-coat scaffold by student’s *t* test.

## Results

### Surface modification on PE substrates

**Figure 1:**
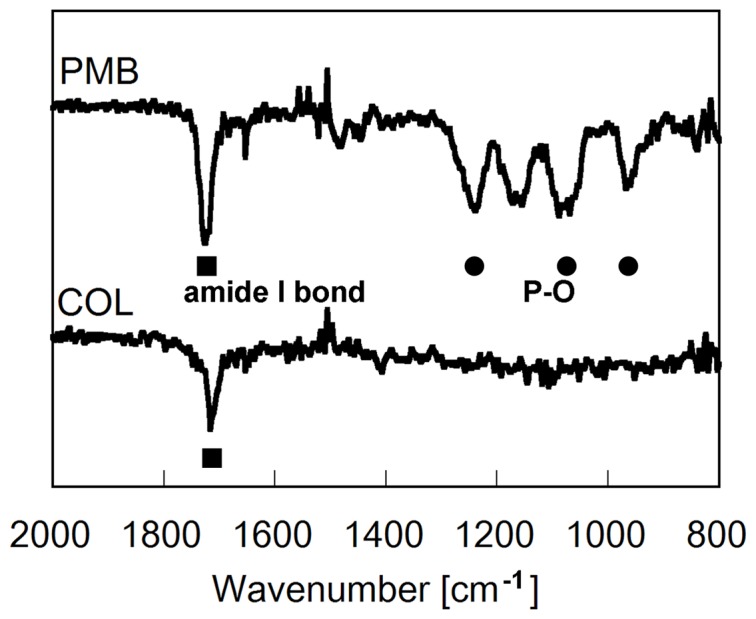
FTIR/ATR spectra of PMB- and collagen-coated PE films.

The static water contact angles of non-, collagen-, and PMB-coated PE films were 102.2±0.6, 63.7±3.3, and 20.4±5.2, respectively. ATR-FTIR spectra of collagen-, and PMB-coated PE films was measured to verify the coating of the PE surface by polymer (Fig. 1). Collagen coating was confirmed from its amine-specific peak observed at 1650 cm^−1^, which is attributed amide-bond. In PMB-coated film, unique absorption peaks at 1240, 1080, and 970 cm^−1^ were observed. These peaks corresponded to the phosphate group (P-O) in the MPC unit in PMB [33]. From these results, it is confirmed that PE scaffold surface can be coated with collagen and PMB successfully.

Because pores in scaffold at mean size of 157 or 32 µm were thought to be too small to penetrate collagen or PMB solution, coating with PMB on PE at the center of scaffold was confirmed by elemental analysis using EDS. The cross-section of the scaffold showed the existence of carbon at the skeletal structure of PE by X-ray mapping. In contrast, phosphorus signals that represents the existence of MPC units were detected at the whole surface of the PMB-coated scaffold. Based on the SEM observations and EDS analysis, it was shown that the polymer coating method was suitable to realize successful covering on the PE scaffold surface even at center of scaffold.

### Scaffold encapsulation

**Figure 2:**
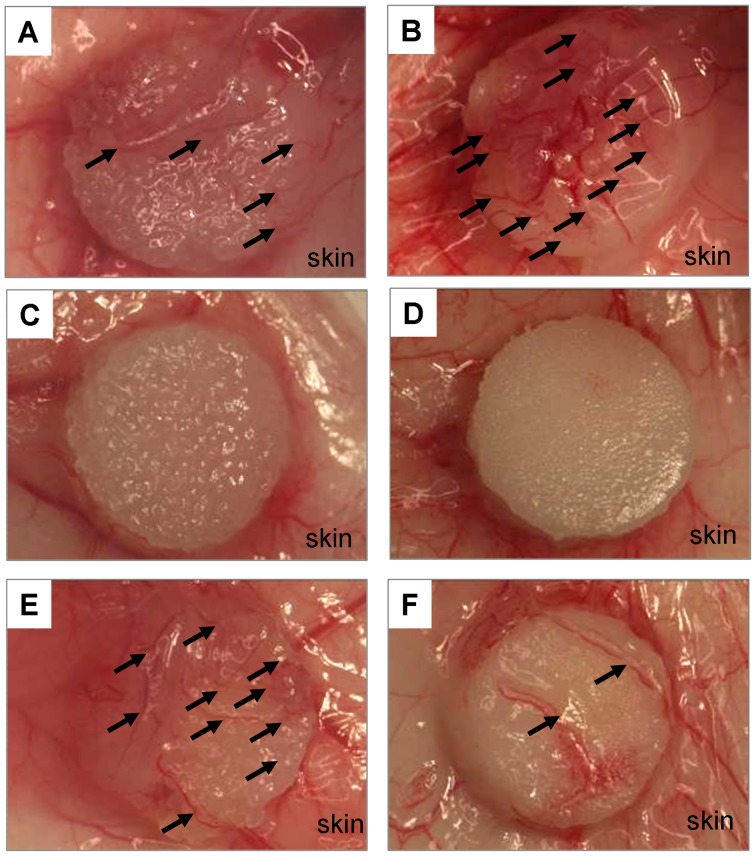
The appearance of scaffolds on day 7 after operation. All scaffolds were attached on the subcutaneous tissue. Scaffolds with a pore size of 157 µm (A, C, and E) and 32 µm (B, D, and F) were implanted. Angiogenesis (arrows) and fibrous tissue encapsulation were compared among non-coat (A and B), PMB-coated (C and D), and collagen-coated (E and F) scaffolds. All scaffold diameters are 6 mm.

**Figure 3:**
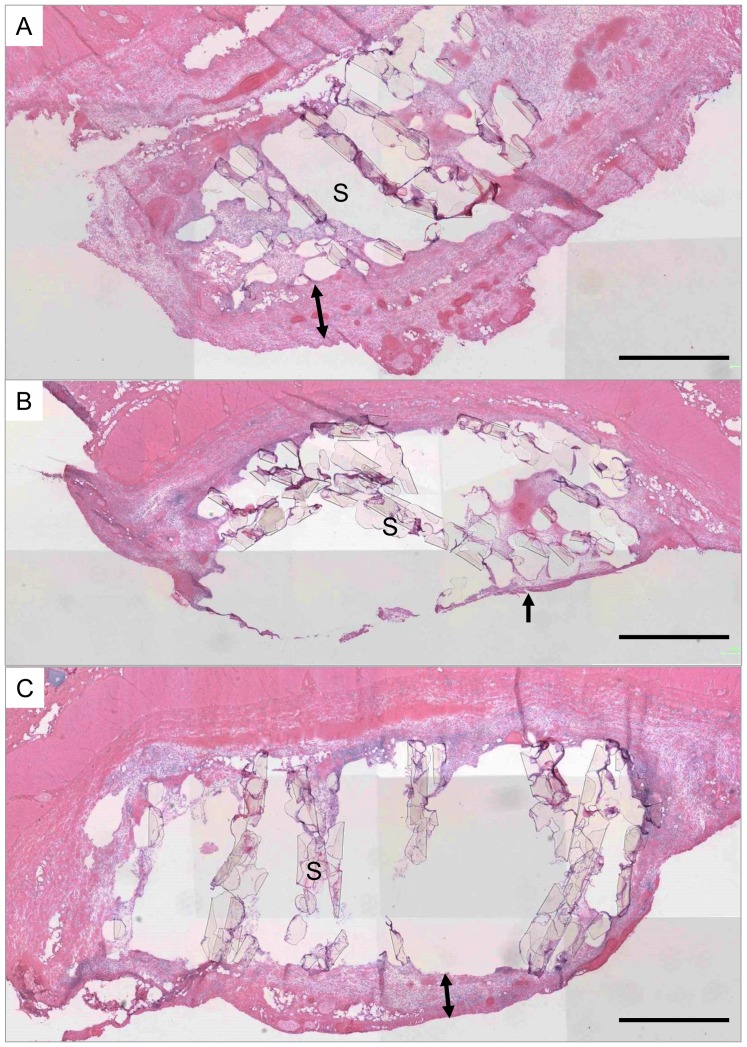
Macroscopic observation of implanted scaffold with non-coat (A), PMB-coated (B), and collagen-coated (C) scaffold which were sliced and stained with HE. Scaffold mean pore size was 157 µm. Scaffolds were attached to rat dorsal skin and encapsulated with layer of fibrous tissue (arrows). Some scaffold skeletons (S) are detached from sliced sample. Bars  = 1 mm.

Six kinds of scaffolds, *i.e.*, non-coat, collagen- and PMB-coated scaffold with 157- and 32-µm pores, were observed at 7 days after implantation in rats. The scaffolds were attached on the subcutaneous tissue as shown in Fig. 2. In observation of both gross and HE staining of cross sectioned scaffold with dorsal skin tissue, fibrous tissue coverage at the periphery of scaffolds were recognized, especially in non-coat and collagen-coated scaffolds (Fig. 2 and Fig. 3). The thickness of encapsulation tissue which is covering non-coat scaffolds was the thickest and that value was 598±80 µm in 157 µm-pored scaffold. On the other hands, encapsulation of PMB-coated scaffolds was very thin and its thickness was 60±12 µm. Collagen-coated scaffold was covered by 300±117 µm. The thickness in PMB- or collagen-coated scaffold was significantly different from that in non-coat scaffold (p<0.01 and p = 0.02, respectively). The difference of encapsulation thickness between PMB- and collagen-coated scaffold was significantly different, too (p = 0.02). However, thickness of encapsulation tissue around 32 µm-pored scaffolds showed different tendency. The respective thickness was 167±120 µm (PMB-coat) and 108±71 µm (non-coat), and 99±14 µm (collagen), and there were no significant difference among three kind of scaffold. Therefore, encapsulation thickness was influenced by not only coating materials but also scaffold pore size. Those results indicated that both materials and shape of scaffold are important for successful tissue regeneration.

### Vessel formation in and around the implanted scaffolds

**Figure 4:**
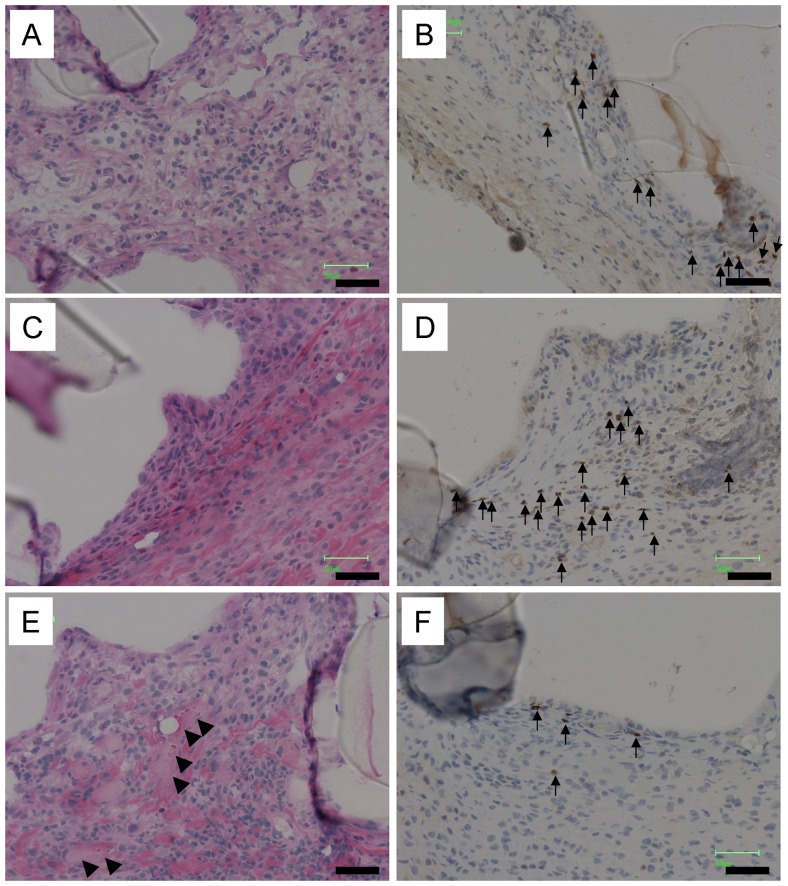
Microscopic histological observations by HE staining (A, C, and E) and CD68 immunostaining (B, D, and F) of non-coat (A and B), PMB-coated (C and D), and collagen-coated (E and F) scaffolds at boundary between scaffold and tissue. Small vessels (arrow heads) and macrophages (arrows) were observed. The mean pore size of the scaffolds was 157 µm. Seven days after operation. Some scaffold skeletons (S) are detached from sliced samples. Bars  = 50 µm.

From the macroscopic observation of scaffolds, small blood vessel formation was observed around the scaffold with both non-coat and collagen-coating (Fig. 2 and Fig. 4). The vessel induction also depended on scaffold pore size and scaffold-coating polymers. For example, more vessels were seen around non-coat scaffolds with 32-µm pore than that with 157-µm pore. On the other hand, more vessels were formed around the scaffold with 157-µm pore than that with 32-µm pore in the collagen-coated scaffold. No microvessels were observed around PMB-coated scaffolds.

The microvessel formations with blood streaming were observed in HE staining of sliced samples, too. High-magnification micrographs at border regions of scaffold and host tissue are shown in Fig. 4 (A, C, and E). Host cells and extracellular matrices were infiltrating into the scaffold pores at the border in every scaffolds. Among them, small vessels towards the inside of scaffold were recognized only in case of collagen-coated scaffold. It was also observed that cells in collagen-coated scaffold showed adhesion on scaffold and spread shapes. On the other hand, cells in the PMB-coated scaffold did not attach to scaffold and showed round shapes.

### Macrophage migration

Border regions of scaffolds were observed after immunostaining for CD68 (Fig. 4 (B, D, and F)). Many CD68-positive cells as monocytes/macrophages were observed in the tissue surrounded the PMB-coated scaffold. On the other hand, the collagen-coated scaffold did not facilitate the migration of CD68-positive cell at the scaffold. Moderate migration of those cells was observed in the tissue surrounded the non-coated scaffold.

### Comprehensive Genetic Analysis

**Figure 5:**
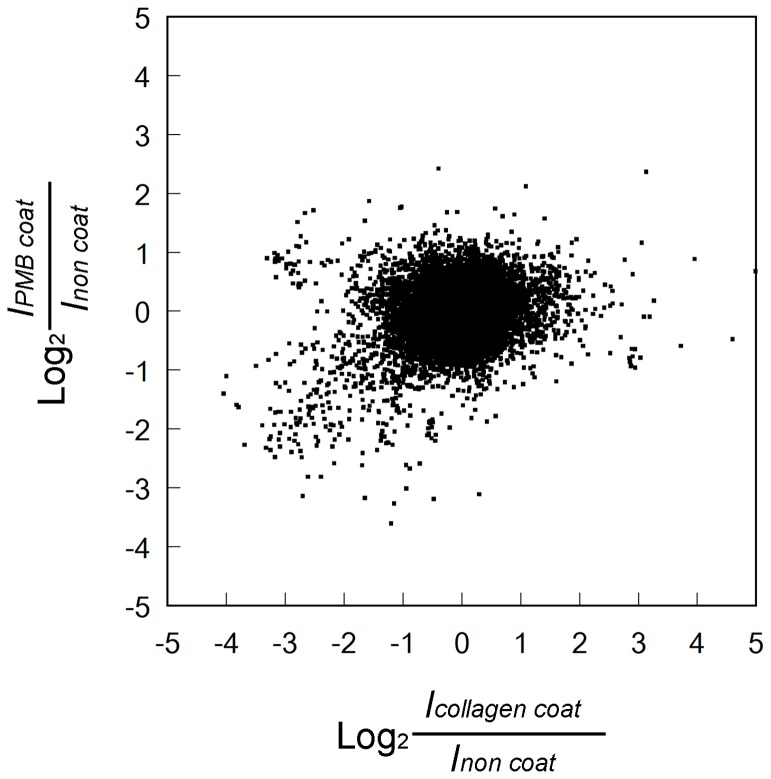
Global analysis of host body responses to PMB-coated and collagen-coated scaffolds. All genes having valid expression levels in non-coated, collagen-coated, and PMB-coated scaffolds were plotted.

The host body reactions at PMB-coated and collagen-coated scaffolds were compared using a DNA microarray assay. Each RNA expression level in cells existing in the scaffold 7 days after implantation was presented as a logarithmic ratio to corresponding levels in the non-coated scaffold. Over half of total genes on the microarray (more than 41K probes per array) presented reliable signals in all scaffolds. In Fig. 5, all of these genes are plotted as a logarithmic ratio (log_2_ ratios) of gene expression on PMB-coated scaffold to that on non-coated scaffold in Y-axis and as a function of that on collagen-coated scaffold to non-coated scaffold in X-axis. The collagen-coated scaffolds showed more widely distributed gene expression (−4< log_2_*I*_collagen coat_/*I*_non coat_ <5) than PMB-coated scaffolds (−2< log_2_*I*_PMB coat_/*I*_non coat_ <2). There were some genes that were up-regulated only in the PMB-coated scaffold. Genes that were up-regulated both in collagen-coated and PMB-coated scaffolds will contain non-specific and material implantation-related host reactions.

**Figure 6:**
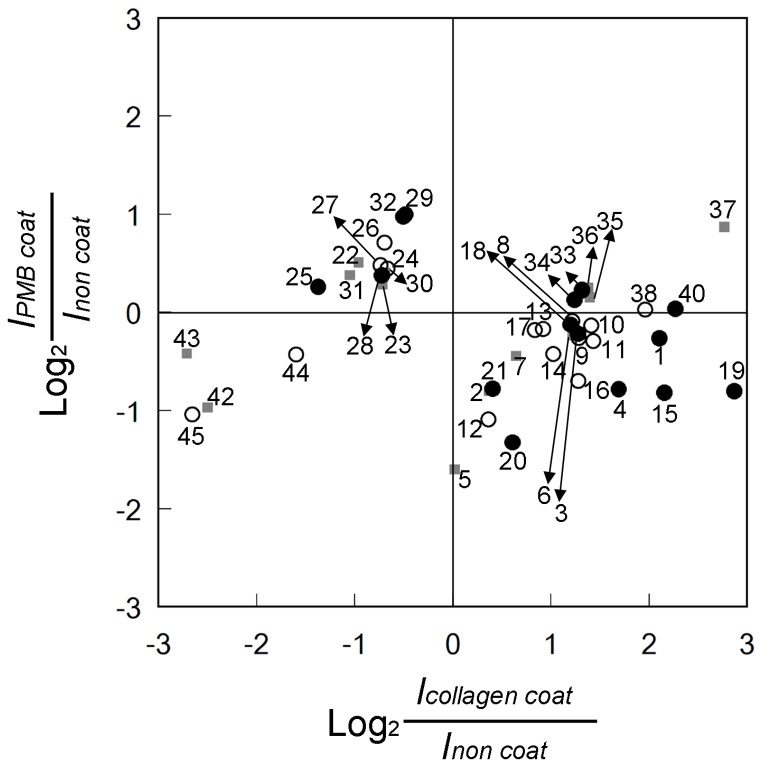
Selected genes that were expressed differently between collagen-coated and PMB-coated and were related to tissue regeneration and inflammation. Closed circle, wound healing promotion factors; Open circle; inflammatory factors; Closed square, uncertain about tissue regeneration.

The number of genes whose expression levels was different more than two-fold between PMB-coated and collagen-coated scaffolds was 1467. Of these, 45 genes were selected by using the keywords related to inflammation, wound healing, angiogenesis, and macrophage polarization. Figure 6 shows relationship of these 45 genes expression between in PMB-coated and in collagen-coated scaffolds. The numbers in Fig. 6 correspond to those in Tables 1–4 and information of each plot is explained in as description of genes in the tables. Functions of those genes involved in inflammation, wound healing, and angiogenesis are indicated, too. Marks “+” and “− “ in the tables show promotion and suppression effect of those genes on the tissue responses. Many genes were in the fourth quadrant, in other words showing down-regulation in PMB and up-regulation in collagen. The number of oppositely regulated genes represented in second quadrant was smaller than the number of genes in the first quadrant, and their expression levels were low. In collagen-specifically up-regulated genes, Th2-type (M2 type) cytokines (MMPs (matrix metallopeptidases)) and collagens, which promote wound healing and angiogenesis to reconstruct tissues, were expressed higher level than inflammation-promoting genes. Among some genes related to angiogenesis that are down-regulated in collagen-coated scaffold, the expression of thrombospondin (THBS) group genes was observed. These included THBS-4, which was actively down-regulated in collagen and suppressed slightly in PMB, and THBS-3, which was down-regulated in collagen and up-regulated in PMB.

**Table 1:** Descriptions and expression levels of genes which were up-regulated in collagen and down-regulated in PMB.

No. in Fig. 6	Description	Inflammation	Wound Healing	Angiogenesis	Relative Expression Level		Ratio
					collagen	PMB	collagen/PMB
up-regulation in collagen and down-regulation in PMB							
1	arginase type II (Arg2)		+		2.102	−0.261	2.362
2	B-cell translocation gene 2, anti-proliferative (Btg2)				0.367	−0.800	1.167
3	collagen and calcium binding EGF domains 1 (Ccbe1)				1.275	−0.216	1.490
4	CD163 molecule-like 1 (CD163l1)		+		1.688	−0.781	2.469
5	collagen, type XI, alpha 1 (Col11a1)				0.018	−1.599	1.616
6	collagen, type XII, alpha 1 (Col12a1)				1.218	−0.233	1.451
7	insulin-like growth factor 1 (IGF1), transcript variant 1				0.644	−0.443	1.087
8	interleukin 1 alpha (IL1a)	+			1.216	−0.090	1.306
9	interleukin 1 beta (IL1b)	+		+	1.285	−0.263	1.548
10	interleukin 1 receptor, type II (IL1r2)	+			1.410	−0.135	1.545
11	interleukin 1 receptor accessory protein (IL1rap), transcript variant 2	+			1.429	−0.293	1.722
12	interleukin 1 receptor antagonist (IL1rn), transcript variant 2	+			0.364	−1.092	1.456
13	interleukin 2 receptor, beta chain (IL2rb)	+			0.920	−0.170	1.089
14	interleukin 2 receptor, gamma chain (IL2rg)	+			1.026	−0.426	1.451
15	interleukin 8 receptor, beta (IL8rb)	+			2.153	−0.818	2.971
16	interleukin 18 receptor 1 (IL18r1)	+			1.278	−0.702	1.980
17	interleukin 18 receptor accessory protein (IL18rap)	+			0.836	−0.180	1.016
18	matrix metallopeptidase 8 (MMP8)		+	+	1.198	−0.123	1.320
19	matrix metallopeptidase 9 (MMP9)		+	+	2.867	−0.802	3.669
20	matrix metallopeptidase 10 (MMP10)		+	+	0.605	−1.323	1.928
21	vascular endothelial growth factor A (VEGF-A), transcript variant 1			+	0.402	−0.778	1.180

Numbers in Fig. 6 correspond to those in the table. Plus and minus in this table indicate promotion and suppression, respectively.

**Table 2:** Descriptions and expression levels of genes which were down-regulated in collagen and up-regulated in PMB.

No. in Fig. 6	Description	Inflammation	Wound Healing	Angiogenesis	Relative Expression Level		Ratio
					collagen	PMB	collagen/PMB
down-regulation in collagen and up-regulation in PMB							
22	collagen, type XIV, alpha 1 (Col14a1)		+		−0.962	0.512	−1.474
23	collagen, type V, alpha 3 (Col5a3)		+		−0.718	0.287	−1.005
24	fibroblast growth factor 10 (Fgf10)		+		−0.690	0.403	−1.093
25	interleukin 10 (IL10)	−	−		−1.376	0.262	−1.638
26	interleukin 17D (IL17d)	+			−0.697	0.711	−1.407
27	interleukin 17 receptor D (IL17rd)	+			−0.665	0.445	−1.110
28	peroxisome proliferator activated receptor gamma (Pparg), transcript variant 2				−0.728	0.379	−1.107
29	procollagen C-endopeptidase enhancer 2 (Pcolce2)				−0.485	1.001	−1.486
30	thrombospondin 3 (TBS3)			−	−0.739	0.481	−1.219
31	transforming growth factor alpha (TGFa)		+		−1.051	0.382	−1.433
32	vascular endothelial growth factor C (VEGF-C)			+	−0.508	0.977	−1.485

Numbers in Fig. 6 correspond to those in the table. Plus and minus in this table indicate promotion and suppression, respectively.

**Table 3:** Descriptions and expression levels of genes which were up-regulated both in collagen and PMB.

No. in Fig. 6	Description	Inflammation	Wound Healing	Angiogenesis	Relative Expression Level		Ratio
					collagen	PMB	collagen/PMB
up-regulation both in collagen and PMB							
33	collagen, type IV, alpha 1 (Col4a1)		+	+	1.313	0.233	1.081
34	collagen, type IV, alpha 2 (Col4a2)		+	+	1.312	0.227	1.085
35	collagen, type XXIII, alpha 1 (Col23a1)				1.392	0.155	1.236
36	disintegrin-like and metallopeptidase (reprolysin type) with thrombospondin type 1 motif, 7 (Adamts7)				1.378	0.254	1.125
37	epidermal growth factor receptor pathway substrate 15 (Eps15)				2.765	0.872	1.893
38	interleukin 1 family, member 9 (IL1f9)	+			1.960	0.029	1.932
39	interleukin 4 induced 1 (IL4i1)		+		1.236	0.130	1.107
40	interleukin 13 receptor, alpha 2 (IL13ra2)		+		2.265	0.038	2.227
41	platelet-derived growth factor, C polypeptide (PDGF-C)				4.987	0.677	4.310

Numbers in Fig. 6 correspond to those in the table. Plus and minus in this table indicate promotion and suppression, respectively.

**Table 4:** Descriptions and expression levels of genes which are up-regulated in collagen and down-regulated in PMB.

No. in Fig. 6	Description	Inflammation	Wound Healing	Angiogenesis	Relative Expression Level		Ratio
					collagen	PMB	collagen/PMB
down-regulation both in collagen and PMB							
42	collagen, type VIII, alpha 2 (Col8a2)				−2.501	−0.967	−1.534
43	disintegrin-like and metallopeptidase (reprolysin type) with thrombospondin type 1 motif, 19 (Adamts19)			−	−2.714	−0.419	−2.295
44	interleukin 11 (IL11)	+			−1.595	−0.430	−1.166
45	thrombospondin 4 (THBS4)			−	−2.657	−1.040	−1.617

Numbers in Fig. 6 correspond to those in the table. Plus and minus in this table indicate promotion and suppression, respectively.

## Discussion

The host body reactions to various biomaterial surfaces in the early period after transplantation affect seriously the following tissue regeneration. Although the details of these reactions have been investigated, the reactions determining the success or failure of tissue regeneration remain unclear. This study revealed important factors for successful tissue regeneration using a comprehensive analysis technique, which has never before been used to analyze tissue regeneration *in vivo*. In this study, we used collagen and PMB whose applications have been investigated already in clinical field, as representative bioactive and bio-inert materials.

Macrophages actively work as an initiator of inflammation and tissue regeneration. Even though the PMB suppressed adhesion and responses of the immune cells *in vitro* [38]–[40], a significant number of monocytes/macrophages were observed by immunostaining of the tissue surrounded PMB-coated scaffold. DNA microarray assay revealed that a number of genes may be actively involved in neglecting the PMB-coated scaffold (Fig. 6, No. 22−32). These results suggest that macrophages may also play a significant role in host body suppressing reactions.

Many interleukins (ILs) were found to be expressed differently in PMB-coated and collagen-coated scaffold. Some IL expressions were specifically up-regulated in PMB-coated scaffold, and those cytokines may interestingly modulate bio-inertness of the materials. Cytokines that were expressed differently in PMB and collagen were classified into four different categories on the basis of their function as promoters or regulators of inflammation and wound healing. The specific up-regulation of IL-1β (Fig. 6, No.9) and IL-10 (Fig. 6, No. 25) was found in collagen-coated scaffold and PMB-coated scaffold, respectively (Table 1–4). These are very distinctive cytokines because IL-1β promotes both inflammation and wound healing processes, which are in conflict with each other, while IL-10 suppresses both of them [12]. The results of histological observation and microarray assay suggest that the induction of both inflammation and wound healing processes may be indispensable for successful tissue regeneration. On the other hand, bio-inert reactions require active suppression of both inflammation and wound healing processes. Chronic inflammation including fibrous encapsulation, may occur as a result of activation of only inflammation or wound healing process.

A blood supply is indispensable to cell survival. Angiogenesis takes place not only in embryonic development or inflammation but also in unusual tissue formation such as tumor development since these cells need new vessels to be fed with oxygen and nutrients [41]. Tissue regeneration also requires angiogenesis in the scaffold. Many researchers have tried to construct blood vessels to regenerate bigger tissue. The construction of blood vessels before target tissue regeneration has been attempted in order to ensure a faster blood supply in regenerating tissues [42]. Others have tried to assess microvessel formation in the porous scaffold by time-course observations [43]. Schutte *et al.* have focused on the importance of blood vessel formation in regenerative tissue in the scaffold, and they have defined VEGF (vascular endothelial growth factor) as anti-inflammatory/pro-wound healing factors in their research [15]. In the present study, two different pore sizes were used, and the angiogenesis behavior was investigated. The tubular formations filled with erythrocytes were observed only in the inner pore of collagen-coated porous scaffold with the pore size of 157 µm (Fig. 4E). It was reported that scaffolds with a smaller pore (20−75 µm) do not induce tissue regeneration because an appropriate angiogenesis is suppressed in and around the scaffolds [43]. In another reports, scaffold with 60 µm mean pore induced more microvessel formation in the scaffold than 5 or 700 µm [32]. In our study, numerous small vessels were observed around non-coated scaffolds and that scaffolds also induced thick fibrous encapsulation (Fig. 4B). Therefore, the formation of many small vessels around non-coated scaffold may be considered to be inflammatory vessels, not tissue-constructive vessels. Plasma leakage from vessels, which is known to be a phenomenon of inflammatory angiogenesis, was not investigated in this study [43], [44]. The collagen coating and the pore size of 157 µm may be adequate to induce the successful tissue regeneration with respect to the angiogenesis-inducible and encapsulation-suppressible tendency.

Angiogenesis is induced by many factors, including VEGF (Fig. 6, No. 21). Some angiogenic factors were expressed especially in collagen scaffold implantation. In tumor tissue, Myc gene, an oncogene is activated and triggers the expression and release of the inflammatory cytokine, IL-1β [45], [46]. The IL-1β, in turn, activates metalloproteinases to release extracellular matrix-bound VEGF. The VEGF reaches the endothelial cells, and growth of a vascular network to the tumor tissue is accomplished. Thus, macroscopic observation showed that up-regulation of IL-1β in collagen-coated scaffold (Fig. 6, No. 9) supported expression of MMPs (Fig. 6, No.18−20) and vessel formation in the collagen-coated scaffold (Fig. 4E).

THBS is the protein, which is released from thrombin-treated platelets [47], [48]. Currently, 5 members of the THBS family have been identified, and THBS-1 and THBS-2 are recognized as anti-angiogenic factors that regulating proliferation and adhesion of endothelial cells [49]. The expression levels of THBS-3 (Fig. 6, No. 30) and THBS-4 (Fig. 6, No.45) were significantly different between collagen-coated and PMB-coated scaffolds and down-regulated in collagen-coated scaffold implantation. Detailed functions of these THBSs are under investigation, and there are no obvious result that explains the influence on endothelial cells. It has been reported that a variant of human THBS-4 that has a proline rather than an alanine at residue 387 suppresses the proliferation and adhesion of cultured endothelial cells [50]. The same as THBS-1 and THBS-2, THBS-3 and THBS-4 contain a type III repeat domain that has been reported to inhibit binding of FGF-2 to endothelial cells, which leads to endothelial cell proliferation *in vitro* [51]. The binding of integrin α_v_β_3_ and α_IIb_β_3_ to the domain regulates the inhibition of endothelial cell adhesion or migration; THBS-4 is therefore considered to have anti-angiogenic function. Synthetic heteroarotinoid, SHetA2, has an anti-cancer function by inhibiting vessel formation [52], [53]. In ovarian cancer cell cultures, gene expression and production of THBS-4 is dramatically increased by the addition of SHetA2 [54]. Those findings suggest that inhibition of angiogenesis is closely related to up-regulation of THBS-4. The down-regulation of THBSs in a collagen-coated scaffold will thus lead to up-regulation of angiogenesis.

Expression of matrix metallopeptidases (MMPs; Fig. 6, No. 18-20) and collagens (Fig. 6, No. 5−6, 22−23, 33−35, and 42) is also modulated by scaffolds. These factors are related to the destruction and reconstruction of tissues. Tissue regeneration requires the destruction of scaffold matrix, reconstruction of extracellular matrices, and cellular arrangement including angiogenesis. Dominant up-regulation of this genes in collagen-coated scaffold was observed in the microarray as same as up-regulation of other angiogenic factors such as VEGF. In a comprehensive manner, collagen-coated scaffold induces up-regulation of angiogenenic and tissue regenerating genes.

Gene expression analysis using microarray give us too many information to understand what is happening. To confirm specific genes that indicate implanted materials fate, narrowing down the focused genes and repeat the measurement of gene expression level must to be performed in the future.

## Conclusion

Even in the presence of advanced technologies of molecular biology, host body reactions to biomaterials using microarray have never been studied. *In vivo* study to understand the reactions is necessary to design a scaffold for successful tissue regeneration. Collagen and the MPC polymers including PMB are clinically used materials who have opposite characteristics. In this study, a microarray experiment was performed using non-coated scaffold as a control material. Other controls such as native tissue and wound healing tissue will be needed to better understand proper tissue regeneration. We narrowed down tissue regeneration-relating genes based on some keywords (inflammation, wound healing, and angiogenesis) which are thought to be indispensable for tissue formation. Bio-inert scaffold slightly up-regulated genes which are related to suppression of inflammation and wound healing. In contrast, a number of genes related to inflammation, wound healing, and angiogenesis were up-regulated in the collagen scaffold. Up-regulation of interleukin-1β and the angiogenesis-relating genes inside the porous scaffolds are the possibly important factors for controlling tissue reconstruction.
